# LncRNA AP000695.2 promotes glycolysis of lung adenocarcinoma via the miR-335-3p/TEAD1 axis: The role of AP000695.2 in glycolysis of lung adenocarcinoma

**DOI:** 10.3724/abbs.2023227

**Published:** 2023-09-18

**Authors:** Shuoyan Xu, Zhiming Cheng, Bulin Du, Yao Diao, Yaming Li, Xuena Li

**Affiliations:** Department of Nuclear Medicine the First Hospital of China Medical University Shenyang 110001 China

**Keywords:** long non-coding RNA, aerobic glycolysis, TEAD1, ceRNA

## Abstract

AP000695.2 is a novel long non-coding RNA (lncRNA). Its aberrant high expression is remarkably associated with poor prognosis of patients with lung adenocarcinoma (LUAD). However, its role and underlying mechanism in LUAD remains unclear. Previous bioinformatics analysis indicated that AP000695.2 may be closely related to the glycolysis of LUAD. This study aims to verify and explore the mechanism of AP000695.2 in glycolysis of LUAD. Overexpression plasmid and siRNA are used to construct cell models of upregulation and downregulation of AP000695.2, respectively. AP000695.2 is highly expressed in lung cancer cell lines as revealed by qPCR. Western blot analysis, FDG uptake, lactate production assay and ECAR determination results show that high expression of AP000695.2 facilitates glycolysis of LUAD cells. CCK-8, EdU staining, Transwell and wound healing assays show that high expression of AP000695.2 promotes cell growth and migration of LUAD. The relationship between AP000695.2 and miR-335-3p is confirmed by bioinformatics analysis and dual-luciferase reporter assays. Through the dual-luciferase reporter assay, TEA domain transcription factor 1 (TEAD1) is identified as a target gene of miR-335-3p. Rescue experiments are applied to verify the relationship among AP000695.2, miR-335-3p and TEAD1. Our study indicates that AP000695.2 is involved in the mechanism of LUAD through functioning as a ceRNA to competitively sponge miR-335-3p, thereby regulating the expression of TEAD1. In the *in vivo* models, AP000695.2 depletion restrains tumor growth and glycolysis. AP000695.2 promotes the glycolysis of LUAD by regulating the miR-335-3p/TEAD1 axis, and it may serve as a potential target of anti-tumor energy metabolism therapy.

## Introduction

According to the latest data of the American Cancer Society, the morbidity and mortality rates of lung cancer are among the highest in both males and females [1], among which lung adenocarcinoma (LUAD) is the most common histological subtype [2]. With the rapid development of molecular biology, great progress has been made in the study of the mechanism of lung cancer, but there are still many unknown factors in the occurrence, development and prognosis of lung cancer. Therefore, elucidating the underlying mechanism to develop innovative therapeutic targets to improve survival rates in patients with LUAD is critical.

Long non-coding RNAs (lncRNAs), which are transcripts with a length of more than 200 nucleotides and little protein-coding ability, have been identified as new regulators of cancer progression [3]. Additionally, it acts as an important regulator for various biological processes, such as cell growth, cell migration, cell invasion and metabolic reorganization [4]. Moreover, abnormally expressed lncRNAs are considered potential alternative biomarkers or therapeutic targets for non-small cell lung cancer (NSCLC) [5]. Therefore, in-depth research exploring new regulatory targets is crucial. In the previous bioinformatics analysis, the Cancer Genome Atlas (TCGA) database suggests that AP000695.2 can be related to the malignant prognosis of various cancers. For example, some studies speculate that AP000695.2 may be involved in the construction of prognosis models of the gastric cancer [ 6‒ 8] and lung adenocarcinoma [9]. However, the role and underlying of AP000695.2 in LUAD remains unclear and deserves further study.

‘Aberrant energy metabolism’ is one of the characteristics of tumor cells [10]. Even under aerobic conditions, tumor cells still tend to produce energy by glycolysis, which is known as the ‘Warburg effect’ [11]. Cancer biology research using genomics showed that the synergy of oncogenes and the activation of downstream pathways exacerbate metabolic disorders [12]. Therefore, the study of regulatory factors targeting glycolytic enzymes can provide new directions for the treatment of malignant progression of LUAD. Existing studies have shown that lncRNAs play an important role in regulating aerobic glycolysis in NSCLC, showing broad research prospects [ 13 , 14]. However, the role of AP000695.2 in glycolysis of LUAD remains unexplored.

Competitive endogenous RNA (ceRNA) is generally regarded as an important mechanism for lncRNAs to participate in the process of tumor biological regulation [ 15, 16]. Small non-coding RNA such as microRNAs (miRNAs) can participate in the regulation of biological behaviors of various tumor cells [17]. TCGA, TargetScan and miRDB database screenings suggest that AP000695.2 may bind to and sequester miR-335-3p to elevate the expression of TEA domain transcription factor 1 (TEAD1). This study aimed to validate the theory and provide a potential target for anti-tumor energy metabolism therapy.

## Materials and Methods

### Bioinformatics analysis

**Figure 1:**
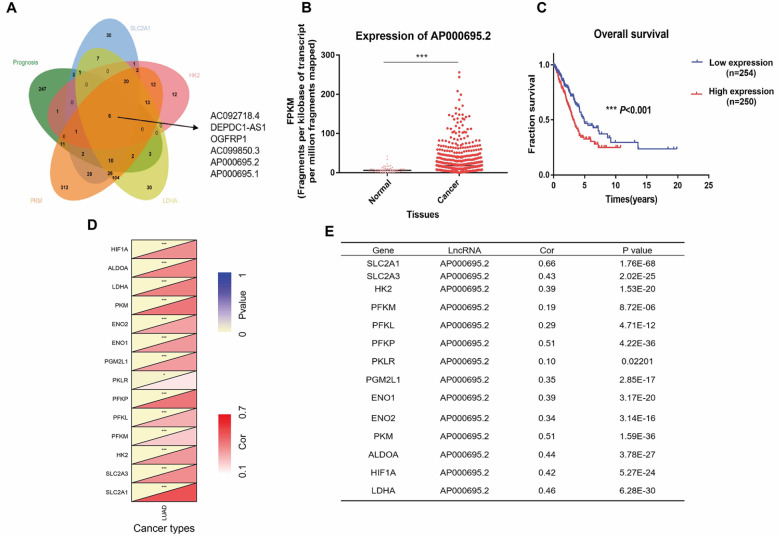
Figure 1 The expression of AP000695.2 in The Cancer Genome Atlas database (TCGA) (A) Six lncRNAs that are correlated with classic glycolysis genes (SLC2A1, HK2 , PKM and LDHA) and have prognostic effects were screened. (B) Bioinformatics analysis showed that the expression of AP000695.2 in 535 cancer tissues is significantly higher than that in 59 normal tissues. (C) The high expression of AP000695.2 is closely related to the poor prognosis. (D) Spearman correlation analysis showed that AP000695.2 is positively correlated with the mRNA expression of various glycolysis-related genes. (E) Correlation coefficient and P value between AP000695.2 and mRNA expression of glycolysis-related genes. Cor: correlation coefficient. ***P<0.001.

Transcriptome data and clinical information were downloaded from the TCGA-LUAD database ( https://portal.gdc.cancer.gov/), including 535 tumor samples and 59 normal samples. The expression data of mRNA and lncRNA were obtained using Strawberry Perl (version 5.32.1.1-64bit), and the ‘limma’ package in the R project (version 4.1.1) was used to obtain lncRNAs with significant differences between tumor and normal samples. The expression data of glycolysis-related genes was extracted, and lncRNAs with significant correlation with the genes (|r| ≥ 0.3, *P*<0.001) were obtained. The clinical data were extracted, and the lncRNAs with a significant difference from the prognosis ( *P*<0.05) were obtained using the ‘survival’ package. Kaplan-Meier (K-M) method and uniCox method were used to screen out lncRNA with significant difference in prognosis. Six lncRNAs were found to be associated with the glycolytic genes ( *SLC2A1*, *HK2*, *PKM* and *LDHA*) and those affecting prognoses were further screened ( Figure 1A). Combined with previous literature [ 6‒ 9], the novel lncRNA-AP000695.2 was selected for this study.

### Cell culture and transfection

A549, H1299, HCC827, PC9, H292, H460, 95D, HBE and HEK-293FT cell lines were purchased from the Cell Bank of Chinese Academy of Sciences (Shanghai, China). All these cells were cultured in Dulbecco’s modified Eagle’s medium/F-12 medium (DMEM/F12; Gibco, Carlsbad, USA) supplemented with 10% fetal bovine serum (FBS; BI, Kibbutz Beit Haemek, Israel) at 37°C with 5% CO _2_. AP000695.2 overexpression plasmid (Sangon Biotech, Shanghai, China) and si-AP000695.2 (Synbio-tech, Suzhou, China) were used to regulate AP000695.2 expression. TEAD1 overexpression plasmid (Origene, Rockville, USA) and si-TEAD1 (target sequence: 5′-GGATCCTCACAAGACGTCA-3′; RiboBio, Guangzhou, China) were used to regulate TEAD1 expression. miR-335-3p mimics, negative control (NC), miR-335-3p inhibitor and inhibitor NC (Synbio-tech) were used for the overexpression and knockdown of miR-335-3p. The transfection of cells was performed using Lipofectamine ^®^ 3000 reagent (Invitrogen, Carlsbad, USA) following the manufacturer’s protocol. Sequences of si-AP000695.2, siRNA NC, miR-335-3p mimics, mimics NC and miR-335-3p inhibitor are listed in Supplementary Table S1.

### Fluorescent *in situ* hybridization (FISH)

A549 and H1299 were seeded into 24-well plates, covered with cell-climbing slices and cultured to 60% confluence. Subsequently, they were simply washed with 1×phosphate buffer solution (PBS) and fixed in 4% paraformaldehyde at room temperature for 10 min. Then, the cells were permeabilized in PBS containing 0.5% Triton X-100 at 4°C for 5 min, and washed with PBS for 5 min. A total of 200 μL of pre-hybridization buffer was added and incubated at 37°C for 30 min. Hybridization was performed overnight using a FISH probe (RiboBio) at 37°C in the dark. Cells were washed three times with 4×SSC with 0.1% Tween 20, once with 2×SSC and then once with 1×SSC at 42°C in the dark for 5 min and once with PBS at room temperature. The cells were then stained with DAPI in the dark for 10 min. Human U6 FISH probes and 18S FISH probes were used as the internal controls of nucleus and cytoplasm, respectively. All images were observed under a fluorescence microscope (Nikon, Tokyo, Japan).

### RNA extraction and qPCR

The total RNA of the cell lines was extracted using Trizol (TaKaRa, Kyoto, Japan). The reverse transcription of mRNA to cDNA was performed using the *Evo M-MLV* RT kit with gDNA Clean for qPCR (Accurate Biotechnology, Changsha, China) and its expression was measured using a *PerfectStart* ^TM^ Green qPCR SuperMix kit (TransGen Biotech, Beijing, China). The expression level of miR-335-3p was assessed using *Bulge-Loop* ^TM^ miRNA qRT-PCR Starter Kit (RiboBio). *β-Actin* and *U6* were used as internal controls. miR-335-3p and mRNA expressions were evaluated using a Light Cycler 480 System II (Roche, Rotkreuz, Switzerland), and the 2 ^−ΔΔCT^ method was used for expression calculation. All primers of mRNA were obtained from Synbio-tech, and the primers of miRNA were purchased from RiboBio. Primer sequences used for qPCR are as follows: *AP000695* . *2* forward, 5′-AAGTCACAGTGGCCTTGAAGT-3′, and reverse, 5′-CAGTGCGTATCCTCTTCGGA-3′; *β-actin* forward, 5′-GATTCCTATGTGGGCGAC-3′, and reverse, 5′-TTGTAGAAGGTGTGGTGCC-3′; *TEAD1* forward, 5′-ATGGAAAGGATGAGTGACTCTGC-3′, and reverse, 5′-TCCCACATGGTGGATAGATAGC-3′; *SLC2A1* forward, 5′-GGCCAAGAGTGTGCTAAAGAA-3′, and reverse, 5′-ACAGCGTTGATGCCAGACAG-3′.

### Western blot analysis

Cells were lysed with RIPA lysis buffer supplemented with phenylmethylsulfonyl fluoride (PMSF) and phosphatase inhibitor (Beyotime Biotechnology, Shanghai, China). Bicinchoninic acid (BCA) assay was used to quantify protein concentration. The proteins were separated on a gel prepared using PAGE Gel Fast Preparation Kit (Epizyme, Shanghai, China) and transferred to polyvinylidene fluoride (PVDF) membranes (Millipore, Billerica, USA). Membranes were blocked with 5% (w/v) non-fat dry milk in Tris-buffered saline containing 0.1% Tween-20 (TBST), and then incubated with the following primary antibodies: anti-glucose transporter GLUT1 antibody (ab115730; Abcam, Cambridge, UK), anti-hexokinase II antibody (ab209847; Abcam), anti-TEF1/TEAD-1 antibody (ab133533; Abcam), anti-LDHA antibody (#3582; CST, Danvers, USA), anti-PKM2 antibody (#4053; CST), anti-N-cadherin antibody (#13116; CST), anti-Vimentin antibody (#5741; CST), anti-HIF-1α antibody (BF8002; Affinity Biosciences, Changzhou, China), anti-E-cadherin antibody (ab40772; Abcam) or mouse anti-β actin mAb (TA-09; ZSGB-BIO, Beijing, China). Subsequently, the membranes were incubated with the corresponding secondary antibody at room temperature for 1 h, and the blots were visualized using enhanced chemiluminescence (ECL) reagent.

### Glucose uptake and lactate production

After 48 h of transfection, 148 kBq/mL ^18^F-FDG was added to each well and incubated for 1 h [18]. The supernatant was collected into tubes for collecting radioactive materials (Kangjie, Taizhou, China). After digestion, the cell suspension was collected and transferred into the tubes. The γ counter was used to detect the count per minute (CPM) in the tube, and the background CPM was removed for the results.

Enzyme working solution and chromogenic agent were prepared following the lactic acid assay kit’s (Nanjing Jiancheng, Nanjing, China) instructions. The transfected cell supernatant, enzyme working solution and chromogenic agent were prepared in the ratio of 0.02:1:0.2, respectively, and mixed well. Then, the samples were incubated in a 37°C water bath for 10 min, and the reaction was terminated using a termination solution. Subsequently, absorbance was measured at 530 nm. The lactic acid content was calculated and standardized according to the protein concentration.

### Determination of extracellular acidification rate (ECAR)

The Agilent Seahorse XF Glycolysis Stress Test Kit (Agilent Technologies, Santa Clara, USA) was used to measure glycolytic function in cells. After 48 h of transfection, 5,000 cells per well were plated into the XFp Cell Culture Miniplate (Agilent Technologies) and incubated overnight at 37°C in a 5% CO _2_ humidified atmosphere. Then, cells were washed once with the assay medium (Seahorse XF Base Medium with 2 mM glutamine) and kept in assay medium at 37°C, in a non-CO _2_ incubator for 1 h. After each compound was diluted in assay medium according to manufacture’s protocol, 10 mM glucose, 1 μM oligomycin and 50 mM 2-DG were loaded into the injection ports in the sensor cartridge in sequence. The Aligent Seahorse XFp Extracellular Flux analyzer (102745-100; Agilent Technologies) was used to perform the Seahorse XF Glycolysis Stress Test assay.

Glycolysis=Maximum rate measurement before oligomycin injection–Last rate measurement before glucose injection.

Glycolytic capacity=Maximum rate measurement after Oligomycin injection–Last rate measurement before glucose injection.

Glycolytic reverse=Glycolytic capacity–Glycolysis.

### Cell counting kit-8 (CCK-8) assay and 5-ethynyl-2′-deoxyuridine (EdU) assay

Cells were seeded into 96-well plates (6000–8000/well) after 24 h of transfection. Cell proliferation was assessed using the CCK-8 assay kit (Bimake, Houston, USA), and a microplate reader (Thermo Fisher Scientific, Carlsbad, USA) was used to measure the absorbance at 450 nm.

After 48 h of transfection, EdU was added to A549 and H1299 cells and incubated for 2 h at 37°C. Then, a cell proliferation assay was performed using BeyoClick™ EdU Cell Proliferation Kit with Alexa Fluor 488 (Beyotime Biotechnology) according to the manufacturer’s protocol. Cell nuclei were stained blue and proliferating cells were stained green, which was quantified under a fluorescence microscope.

### Transwell assay and wound healing assay

To detect the cells’ migratory capability, 1×10 ^5^ cells were seeded into a Transwell apparatus (Corning, New York, USA) containing a serum-free medium. A medium containing 20% FBS functioned as a chemoattractant in the lower chamber. After incubation for 18 h, the migrated cells were stained, photographed and counted using a microscope.

After 24 h of transfection, the middle of the well was scratched using a 200-μL pipette tip. After PBS washing, the wells were visualized and photographed under a microscope. Cells were further cultured in serum-free medium for 24 h and subsequently photographed. The healing rate of the cells within 24 h was calculated, which reflected the migratory ability of cells.

### Dual-luciferase reporter assay

Dual luciferase reporter assay was performed using Dual-Lumi™ II Luciferase Reporter Gene Assay Kit (Beyotime Biotechnology) and a fluorescent microplate reader (Thermo Fisher Scientific). All plasmids were constructed by SyngenTech (Beijing, China) and transfected into cells. Their sequences were listed in Supplementary Table S2. After 24 h, the cells were lysed, centrifuged (10,000‒15,000 *g*, 3‒5 min), and the supernatant was retained. A total of 100 μL firefly luciferase detection reagent was added to every 20 μL sample, incubated at room temperature for 5 min and then detected using a fluorescent microplate reader, with the result recorded as fLuc. Subsequently, 100 μL renilla luciferase detection reagent was added to the sample and mixed well. The results were recorded as rLuc using a fluorescent microplate reader. The results were presented as the relative expression levels of fLuc and rLuc (fLuc /rLuc ratios).

### Animal models

Female BALB/c mice, aged 3‒4 weeks, were obtained from Beijing HFK Bioscience Co. Ltd (Beijing, China) and raised in a specific pathogen free (SPF) laboratory. The animal studies were approved by the Institutional Animal Care and Use Committee (IACUC) of China Medical University (CMU2021614). Standard animal care and laboratory guidelines were followed according to the IACUC protocol. Subcutaneous xenograft was established by injecting 1×10 ^7^ cells/mouse H1299 cells into the left armpit of mice. The length (a) and width (b) of the mouse tumors were measured every 3 days using a vernier calliper, and volume (V) was calculated as follows: V=(a×b ^2^)/2. After the tumor volume reached 5 mm×5 mm, 5 nmole si-AP000695.2 or si-NC diluted with 100 μL RNase-free water was injected into the tumor. A total of 5 nmole/time was administered to each mouse, once every three days for two weeks, with a total of 5 times. The si-AP000695.2 for animals (target sequence is 5′-GCAGGAAGATGTACGTGAA-3′) and the negative control were siRNA modified by 2′Ome+5′Chol, which were synthesized by RiboBio.

### Micro-PET examination

Three days after the fifth injection, mice were imaged with micro-PET. The nude mice underwent fasting for at least 6 h before testing. The body length and weight of nude mice were measured. Synthetic ^18^F-FDG (370 kBq/g) imaging agent diluted with normal saline was injected into the tail vein of nude mice. After 45 min for drug absorption, mice were fixed in a prone position on the beds of micro-PET (Madic, Jinan, China). Two beds were collected and scanned for 15 min under isoflurane anesthesia. Reconstruction was performed using 3D-OSEM with the following parameters: physical resolution, 1.3 mm; image resolution, 0.9105 mm (axial position)×0.9 mm (sagittal)×0.9 mm (coronal); sensitivity, 6%; noise equivalent count, 226 kcps. PET images were processed on the Dell Precision T5610 workstation using the Metis viewer software, and the ROIs of the lesion was calculated to obtain SUVmax.

### Immunohistochemistry (IHC) staining

Immunohistochemical analysis was performed following the protocol of UltraSensitive ^TM^ SP (Mouse/Rabbit) IHC kit (MXB Biotechnologies, Fuzhou, China). After deparaffinization, rehydration, antigen retrieval and blocking, the slides were incubated with the primary antibodies of anti-glucose transporter GLUT1 antibody (ab115730; Abcam), anti-hexokinase II antibody (ab209847; Abcam), anti-TEF1/TEAD-1 antibody (#12292; CST), anti-LDHA antibody (#3582; CST) or anti-PKM2 antibody (#4053; CST) at 4°C overnight. After washing, the slides were incubated with the biotin-conjugated second antibody for 10 min at room temperature, followed by incubating with enzyme conjugate HRP (horseradish peroxidase)-streptavidin. Finally, the slides were visualized using a DAB kit (MXB Biotechnologies, Fuzhou, China). All samples were evaluated by two independent pathologists.

### Statistical analyses

SPSS 21.0, GraphPad Prism 7.0, R project and Image J were used for data processing and mapping. Data are expressed as the mean±standard deviation (SD), and count data are presented as the frequency. For variables that did not fit a normal distribution, Mann Whitney U-tests were performed for comparisons. Differences between two groups were evaluated using the Student’s *t*-test when F-test validated the homogeneity of variance or the Welch *t*-test when inhomogeneity of variance existed. All experiments were repeated at least three times. Differences are considered significant at *P* ≤ 0.05.

## Results

### AP000695.2 is upregulated in LUAD tissues and is positively correlated with the expression of glycolytic genes

Bioinformatics analysis showed that AP000695.2 is correlated with the expression of glycolytic genes ( *SLC2A1*, *HK2*, *PKM* and *LDHA*) and the prognosis of LUAD ( Figure 1A). The expression of AP000695.2 in 535 cancer tissues was significantly higher than that in 59 normal tissues ( Figure 1B). Patients with follow-up data were divided into high- ( *n*=250) and low-expression ( *n*=254) groups, with the median expression of AP000695.2 as the boundary. K–M curve indicated that the high expression of AP000695.2 is closely related to the poor prognosis ( Figure 1C). Furthermore, *Spearman* correlation analysis showed that AP000695.2 is positively correlated with the mRNA expression of various glycolysis-related genes ( Figure 1D,E).

### AP000695.2 is upregulated in lung cancer cells

**Figure 2:**
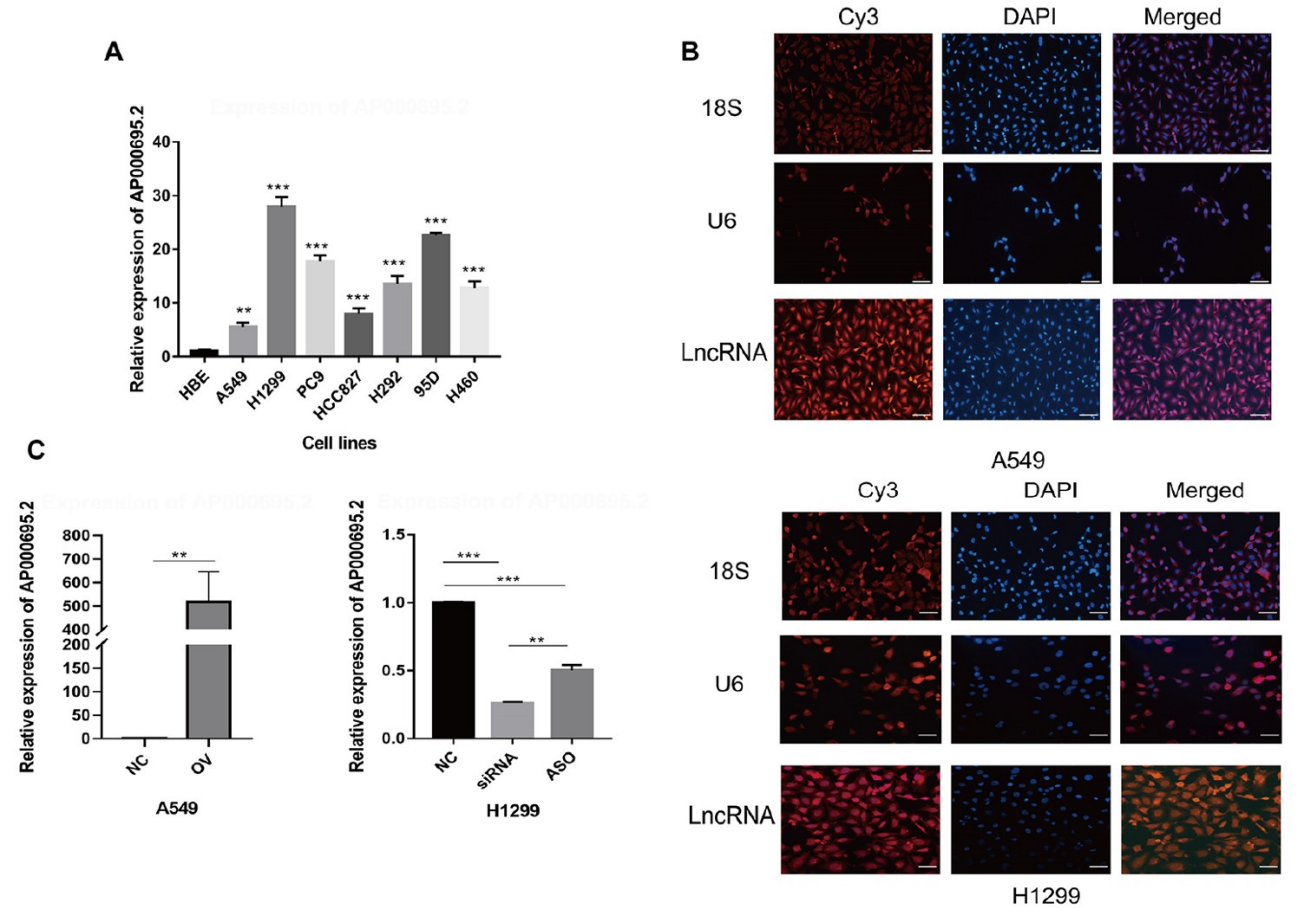
Figure 2 The expression of AP000695.2 in lung cancer cells (A) Quantitative real-time polymerase chain reaction (qPCR) results showed that AP000695.2 was highly expressed in A549, H1299, HCC827, PC9, H292, 95D and H460 cell lines compared with the control group HBE. (B) AP000695.2 is distributed in the nucleus and cytoplasm of A549 and H1299 cells (magnification × 200, scale bar: 100 μm). (C) qPCR results showed that the expression level of AP000695.2 in overexpression group of A549 cell line (OV group) was significantly higher than that in negative control group (NC group) and the expression level of AP000695.2 in the siRNA and ASO groups of H1299 cell line was significantly lower than that in the negative control group (represented by NC group). ** P<0.01, ***P<0.001.

qPCR analysis showed that the expression of AP000695.2 in four LUAD cell lines A549, H1299, HCC827, PC9 and three NSCLC cell lines H292, 95D and H460 were higher than those in the control group HBE ( Figure 2A). Therefore, in the follow-up experiment, the H1299 cell line showing the highest expression level was selected for downregulation and the A549 cell line showing the lowest expression level was selected for upregulation. The localization of AP000695.2 and U6 in the nucleus and 18S in the cytoplasm of the cell lines were detected by FISH assay, indicating that AP000695.2 was distributed in both the cytoplasm and nucleus ( Figure 2B). Transfection of AP000695.2 overexpression plasmid was used to upregulate the expression of AP000695.2 in the A549 cell line. qPCR results showed that the expression level of AP000695.2 in the overexpression group (OV group) was significantly higher than that in the negative control group (NC group) ( Figure 2C). The expression of AP000695.2 in the H1299 cell line was downregulated by the transfection of siRNA and antisense oligonucleotide (ASO), with AP000695.2 expression in the siRNA and ASO (target sequence: 5′-GCACTGACAAACAACACCCG-3′; RiboBio) groups significantly lower than that in the negative control group (represented by the NC group) (74%±0.3%, 50%±2%). Owing to the higher interference efficiency of siRNA, it was selected for subsequent experiments ( Figure 2 C).

### AP000695.2 promotes LUAD cell glycolysis

**Figure 3:**
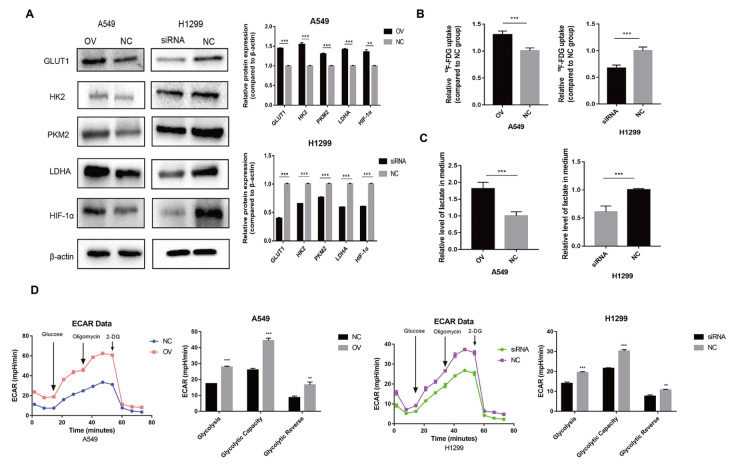
Figure 3 AP000695.2 promotes LUAD cell glycolysis (A) Western blot analysis of the expressions of GLUT1, HK2, PKM2, LDHA and HIF-1α. Compared with NC group, the protein expression levels of GLUT1, HK2, PKM2, LDHA and HIF-1α in A549 cell line OV group were significantly increased. The protein expression levels of GLUT1, HK2, PKM2, LDHA and HIF-1α in H1299 cell line siRNA group were significantly lower than those in NC group. (B) After overexpression of AP000695.2, the uptake rate of 18F-FDG in OV group increased in A549 cells. After the expression of AP000695.2 was downregulated, the uptake rate of 18F-FDG in siRNA group in H1299 cells was decreased. (C) The content of lactic acid after overexpression of AP000695.2 was higher than that of the control group. Compared with the control group, the content of lactic acid was decreased after AP000695.2 was lowered. (D) The level of glycolysis, glycolytic capacity and glycolytic reverse were increased after overexpression of AP000695.2, while they were decreased after downregulation of AP000695.2 expression. *P<0.05, **P<0.01, *** P<0.001.

After upregulation of AP000695.2 expression, the protein expressions of GLUT1, HK2, PKM2, LDHA and HIF-1α in the OV group were significantly higher than those in the NC group, with the expression of β-actin as the control ( Figure 3A). The protein expressions of GLUT1, HK2, PKM2, LDHA and HIF-1α in the siRNA group, which was used to down-regulate the expression of AP000695.2, were significantly lower than those in the NC group, with the expression of β-actin as the control ( Figure 3A). The CPM obtained by detecting ^18^F-FDG using a γ counter can be used to represent the uptake of ^18^F-FDG by cells, indirectly reflecting the glucose uptake level of cells. Cell supernatant and intracellular CPM were measured, and FDG uptake rate was calculated. The relative value of the experimental group was calculated with the NC group as the control. The results showed that after overexpression of AP000695.2, the uptake rate of ^18^F-FDG in the OV group was increased in A549 cells ( Figure 3B). After the downregulation of AP000695.2 expression, the uptake rate of ^18^F-FDG in the siRNA group in H1299 cells was decreased ( Figure 3B). A lactic acid assay kit was used to detect the content of lactic acid in the transfected culture medium, wherein the content of lactic acid after overexpression of AP000695.2 was higher than that of the control group ( Figure 3C). Compared with the control group, the content of lactic acid decreased after AP000695.2 expression was downregulated ( Figure 3C). Next, we evaluated the level of glycolysis, glycolytic capacity and glycolytic reverse by measuring ECAR with the Aligent Seahorse XF Glycolysis Stress Test Kit. The results showed that the level of glycolysis, glycolytic capacity and glycolytic reverse were increased after overexpression of AP000695.2, but decreased after downregulation of AP000695.2 expression ( Figure 3D).

### AP000695.2 facilitates LUAD cell proliferation, migration and EMT processes

**Figure 4:**
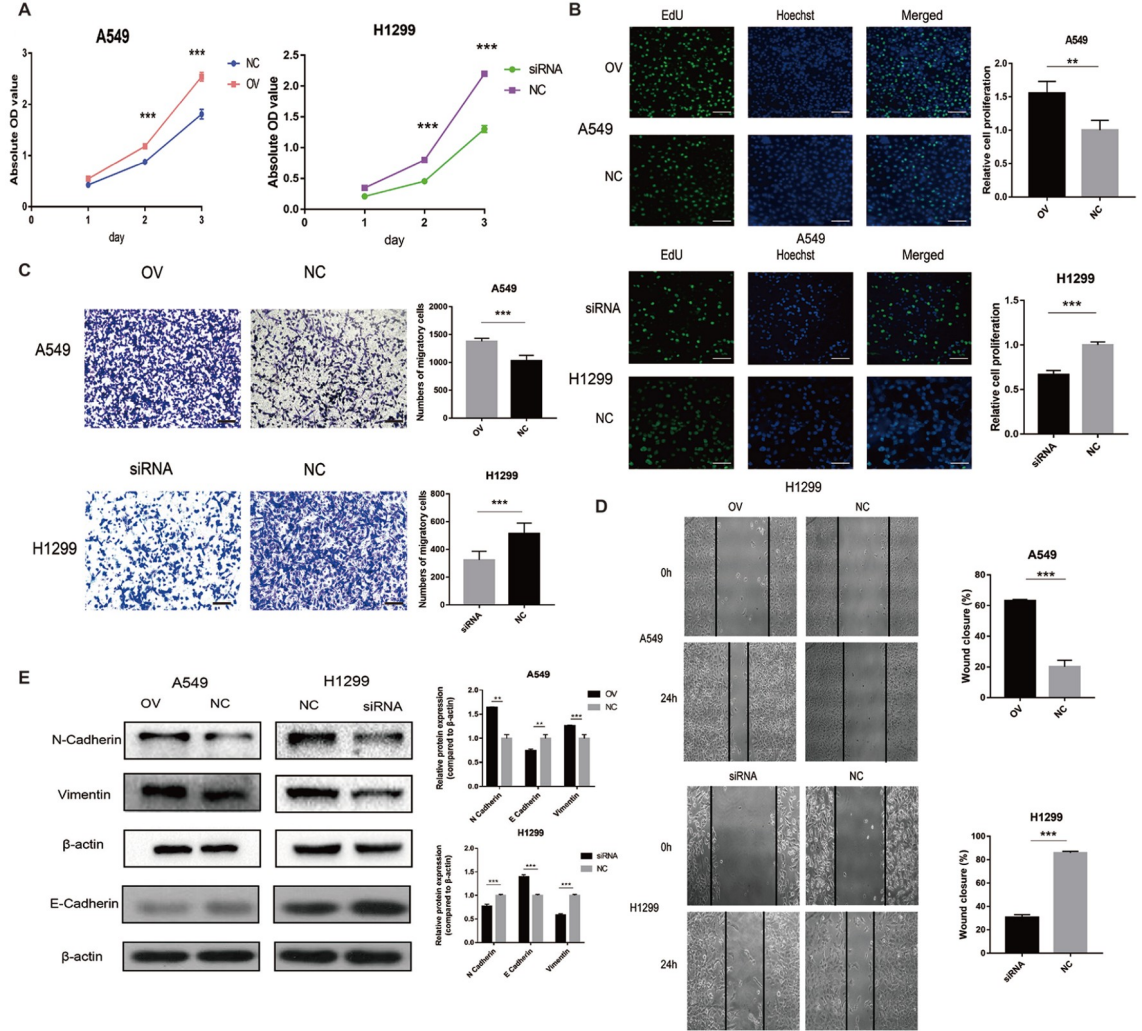
Figure 4 AP000695.2 facilitates lung adenocarcinoma cell proliferation, migration and epithelial–mesenchymal transition (A) The absorbance of the OV group cells in the A549 cell line was higher than that of the negative control (NC) group on days 2 and 3. In the H1299 cell line, the absorbance of cells in the siRNA group was lower than that in the NC group on days 2 and 3. (B) The cell proliferation rate was increased after overexpression of AP000695.2. The proliferation rate of cells was decreased after the downregulation of AP000695.2 (magnification × 200, scale bar: 100 μm). (C) The number of migrated A549 cells of the OV group was higher than that of the NC group. In the H1299 cell line, the number of migrated cells in the siRNA group was lower than that in the NC group (magnification × 200, scale bar: 100 μm). (D) The migration ability of the OV group was stronger than that of the NC group in the A549 cell line. In the H1299 cell line, the cell migration ability of the siRNA group was weaker than that of the NC group (magnification × 100). (E) Western blot analysis of the protein expression levels of N-cadherin, E-cadherin and Vimentin after overexpression and downregulation of AP000695.2, respectively. **P<0.01, ***P<0.001.

The absorbance of cells at 450 nm was detected on days 1, 2 and 3 after transfection. The absorbance of the OV group cells in the A549 cell line was higher than that of the NC group on days 2 and 3 ( Figure 4A). In the H1299 cell line, the absorbance of cells in the siRNA group was lower than that of the NC group at days 2 and 3 ( Figure 4A). EdU-488 cell proliferation detection kit was used to detect the cell proliferation ability. The results showed that the cell proliferation rate was increased after overexpression of AP000695.2 ( Figure 4B). However, the proliferation rate of cells was decreased after downregulation of AP000695.2 ( Figure 4B). Transwell assay detects the number of cells penetrating the polycarbonate membrane, which represents migration ability of cells. The number of migrated A549 cells in the OV group was higher than that in the NC group ( Figure 4C). In the H1299 cell line, cell migration in the siRNA group was lower than that in the NC group ( Figure 4C). The wound healing assay detects the distance of cell migration within 24 h, which also represents cell migration ability. The migration ability of the OV group was stronger than that of the NC group in the A549 cell line ( Figure 4D). In the H1299 cell line, the cell migration ability of the siRNA group was weaker than that of the NC group ( Figure 4D). N-cadherin, E-cadherin and Vimentin are important factors in EMT, with their expressions indicating the presence of EMT. The protein expressions of N-cadherin and Vimentin were increased after AP000695.2 overexpression compared with the expression of β-actin, but they were decreased after downregulation of AP000695.2 expression, compared with the expression level of β-actin, and the difference was statistically significant ( Figure 4E). The protein expression of E-cadherin was decreased after AP000695.2 overexpression compared with the expression of β-actin, and it was increased after downregulation of AP000695.2 expression, compared with the expression level of β-actin, and the difference was statistically significant ( Figure 4E).

### AP000695.2 promotes TEAD1-mediated transcription of GLUT1

**Figure 5:**
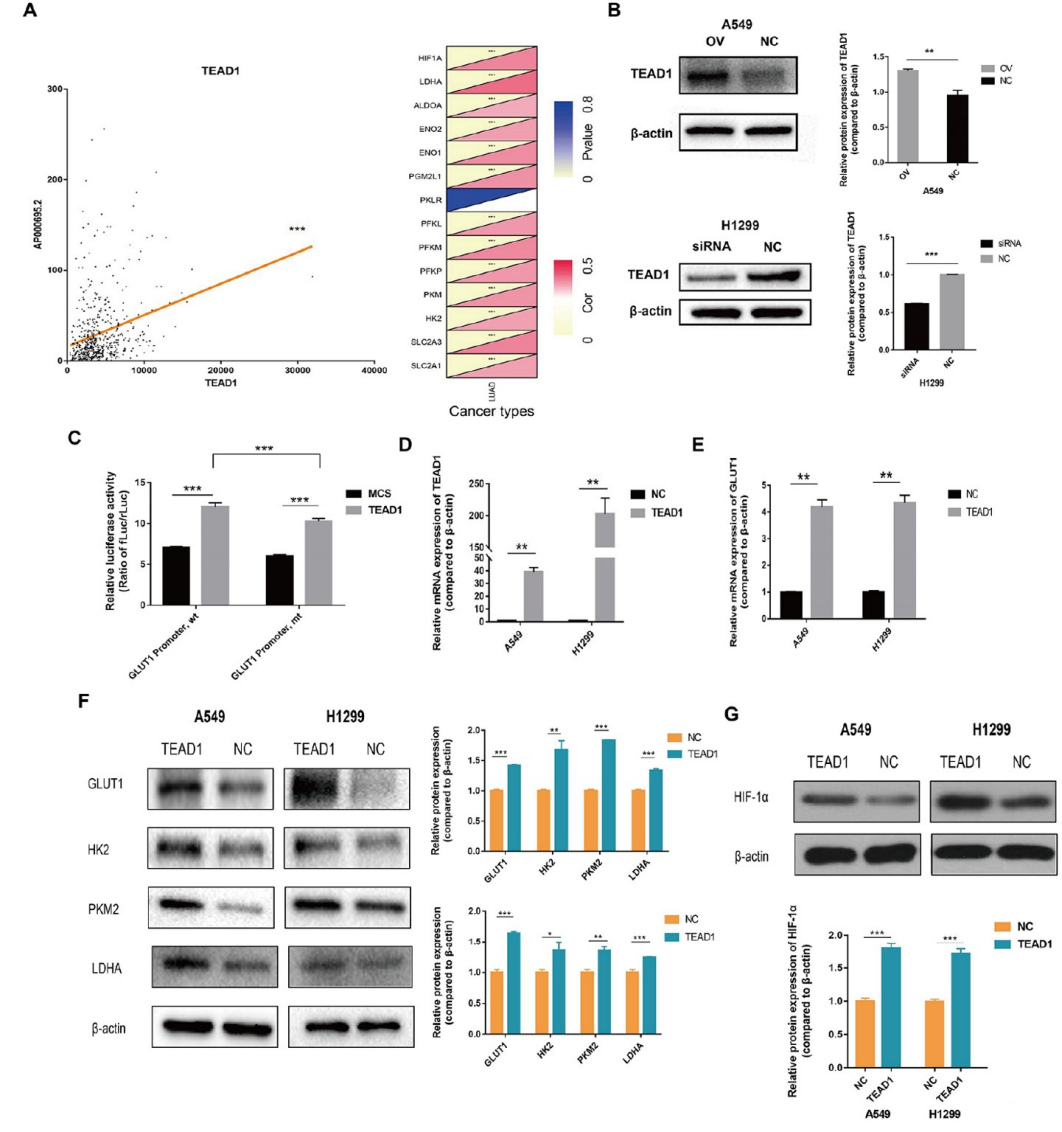
Figure 5 AP000695.2 promotes TEAD1-mediated transcription of GLUT1 (A) Spearman correlation analysis in The Cancer Genome Atlas (TCGA) database showed that there is a positive correlation between AP000695.2 expression and TEAD1 mRNA expression (r = 0.2829). A significant positive correlation between the expression of TEAD1 mRNA and the mRNA expression of various glycolysis-related factors was observed. (B) Cellular experiments showed that the protein expression level of TEAD1 was increased after overexpression of AP000695.2, but decreased after the downregulation of AP000695.2. (C) In the GLUT1 Promoter, wt group, the expression of luciferase was significantly increased after overexpression of TEAD1. In the GLUT1 Promoter, mt group, the expression of luciferase was significantly increased after overexpression of TEAD1, and the expression level of luciferase in GLUT1 Promoter, wt group was significantly higher than that of GLUT1 Promoter, mt group after overexpression of TEAD1. MCS indicates negative control while TEAD1 indicates TEAD1 overexpression plasmid. (D) The expression of TEAD1 in TEAD1 overexpression (TEAD1) group was significantly higher than that in the NC group. (E) qPCR analysis showed that the mRNA expression level of GLUT1 in A549 and H1299 cell lines was increased after upregulating the expression of TEAD1. (F) Western blot analysis showed that the protein expression levels of GLUT1, HK2, PKM2 and LDHA in A549 and H1299 cell lines were increased after upregulating the expression of TEAD1. (G) Western blot analysis showed that the protein expression level of HIF-1α in A549 and H1299 cell lines was increased after upregulating the expression of TEAD1. * P<0.05, **P<0.01, ***P <0.001.

Then, we further explored the mechanism of AP000695.2 regulating LUAD. Spearman correlation analysis in TCGA database showed that there is a positive correlation between AP000695.2 expression and TEAD1 mRNA expression ( Figure 5A, r=0.2829). A significant positive correlation between the expression of TEAD1 mRNA and the mRNA expression of various glycolysis-related factors was observed ( Figure 5A). Therefore, AP000695.2 participates in the regulation of LUAD glycolysis by regulating TEAD1. Cellular experiments showed that the protein expression of TEAD1 was increased after overexpression of AP000695.2 ( Figure 5B), but decreased after the downregulation of AP000695.2 expression ( Figure 5B). JASPAR [19] indicates that there are 12 binding sites between the promoters of *TEAD1* and glucose transporter 1 ( *GLUT1*) (2 kb upstream of the transcription initiation site was selected as the research sequence, with a relative threshold of 85%). The specific locations are shown in Supplementary Table S3. All binding sites are knocked out to form the *GLUT1* mutant promoter. The *GLUT1* wild-type promoter (GLUT1 Promoter, wt) and the *GLUT1* mutant promoter (GLUT1 Promoter, mt) were constructed. In the GLUT1 Promoter, wt group, the expression of luciferase was significantly higher after overexpression of TEAD1, which indicated that TEAD1, a regulatory element, activates the transcription of *GLUT1* promoter. In the GLUT1 Promoter, mt group, the expression of luciferase was significantly higher after overexpression of TEAD1, and the expression level of luciferase in GLUT1 Promoter, wt group was significantly higher than that of GLUT1 Promoter, mt group after overexpression of TEAD1, which indicated that TEAD1 activates the transcription of *GLUT1* promoter even after the mutation of all sites ( Figure 5C).

To further verify the regulatory relationship between TEAD1 and GLUT1, TEAD1 overexpression plasmid and corresponding negative control plasmid were transfected into A549 and H1299 cells, respectively, and the transfection efficiency was detected by qPCR assay. The results showed that TEAD1 expression in the TEAD1 overexpression (TEAD1) group was significantly higher than that in NC group ( Figure 5D). The qPCR experiment showed that the mRNA expression level of GLUT1 in A549 and H1299 cell lines increased after upregulation of TEAD1 expression ( Figure 5E). Western blot analysis showed that the protein expression level of GLUT1 in A549 and H1299 cell lines increased after upregulation of TEAD1 expression ( Figure 5F). Western blot analysis also showed that the protein expression levels of HK2, PKM2, LDHA and HIF-1α in A549 and H1299 cell lines were increased after upregulating the expression of TEAD1 ( Figure 5F,G), suggesting that TEAD1 may activate glycolysis pathway by promoting the expression of GLUT1 and regulate the expression of HIF-1α.

### AP000695.2 promotes LUAD glycolysis, proliferation and migration via TEAD1

**Figure 6:**
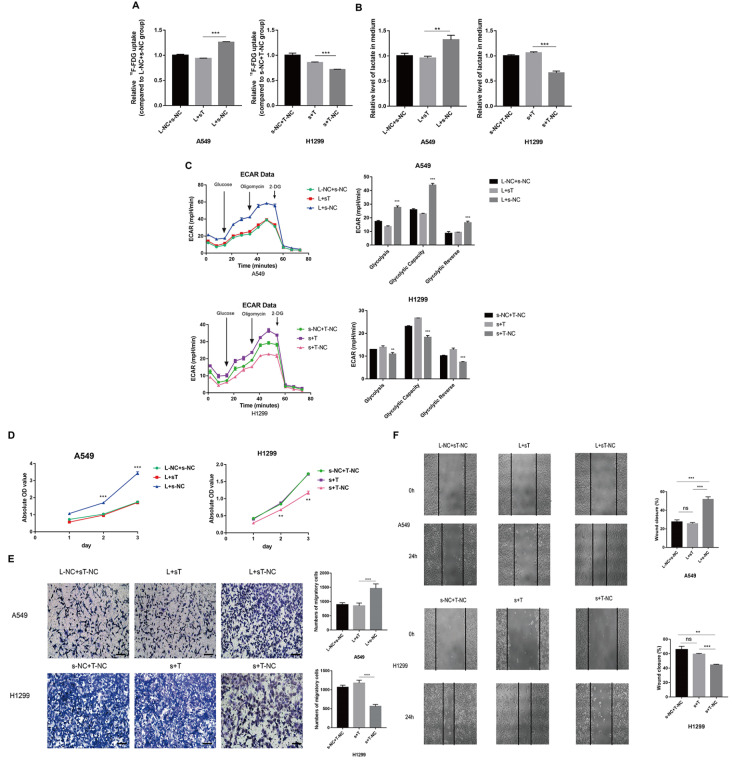
Figure 6 AP000695.2 promotes LUAD glycolysis, proliferation and migration via TEAD1 (A) The relative amount of FDG uptake of the L+sT group (transfected with AP000695.2 overexpression plasmid and TEAD1 siRNA) was lower than that of the L+s-NC (transfected with AP000695.2 overexpression plasmid and TEAD1 siRNA NC) group. The amount of FDG uptake in the s+T (transfected with AP000695.2 siRNA and TEAD1 overexpression plasmid) group was higher than that of the s+T-NC group (transfected with AP000695.2 siRNA and NC of TEAD1 overexpression plasmid). (B) The amount of lactic acid production in the L+sT group was lower than that in the L+s-NC group. The amount of lactic acid produced in the s+T group was higher than that in the s+T-NC group. (C) The level of glycolysis, glycolytic capacity and glycolytic reverse in the L+sT group were lower than those in the L+s-NC group. The level of glycolysis, glycolytic capacity and glycolytic reverse in the s+T group were higher than those of the s+T-NC group. (D) The cell proliferation ability of the L+sT group was lower than that of the L+s-NC group, and that of the s+T group was higher than that of the s+T-NC group on the 2nd and 3rd day after transfection. (E) The cell migration ability of the L+sT group was lower than that of the L+s-NC group, and that of the s+T group was higher than that of the s+T-NC group (magnification×200, scale bar: 100 μm). (F) The cell migration ability of the L+sT group was lower than that of the L+s-NC group, and that of the s+T group was higher than that of the s+T-NC group (magnification×100). **P<0.01, ***P <0.001.

The relative amount of FDG uptake of the L+sT group (transfected with AP000695.2 overexpression plasmid and TEAD1 siRNA) was lower than that of the L+s-NC group (transfected with AP000695.2 overexpression plasmid and TEAD1 siRNA NC). The amount of FDG uptake in the s+T group (transfected with AP000695.2 siRNA and TEAD1 overexpression plasmid) was higher than that of the s+T-NC group (transfected with AP000695.2 siRNA and NC of TEAD1 overexpression plasmid) ( Figure 6A). The lactic acid content in the L+sT group was lower than that in the L+s-NC group in A549 cell, and the lactic acid content in the s+T group was higher than that in the s+T-NC group in H1299 cell ( Figure 6B). The level of glycolysis, glycolytic capacity and glycolytic reverse in the L+sT group was lower than those in the L+s-NC group in A549 cell, while those in the s+T group was higher than those in the s+T-NC group in H1299 cell ( Figure 6C). These results indicated that AP000695.2 is involved in the regulation of cell glycolysis through TEAD1. Hence, AP000695.2 has the potential to participate in the regulation of cell glycolysis through TEAD1. CCK-8 assay was performed on the 1st, 2nd and 3rd day after transfection, which showed that the cell proliferation ability of the L+sT group was lower than that of the L+s-NC group, and that of the s+T group was higher than that of the s+T-NC group ( Figure 6D). This indicates that AP000695.2 is involved in cell proliferation regulation through TEAD1. Cells were seeded in a Transwell chamber on the 1st day after transfection, and the number of cells penetrating the polycarbonate membrane was observed by staining after 18 h. The results showed that the cell migration ability of the L+sT group was lower than that of the L+s-NC group, and that of the s+T group was higher than that of the s+T-NC group ( Figure 6E). Wound healing assays were performed on the 1st day after transfection, and photographs were taken at 0 h and 24 h. The cell migration ability of the L+sT group was lower than that of the L+s-NC group, and that of the s+T group was higher than that of the s+T-NC group ( Figure 6F), indicating that AP000695.2 participates in the regulation of cell migration via TEAD1.

### miR-335-3p acts as a competing endogenous RNA participating in the regulation of TEAD1 and AP000695.2

**Figure 7:**
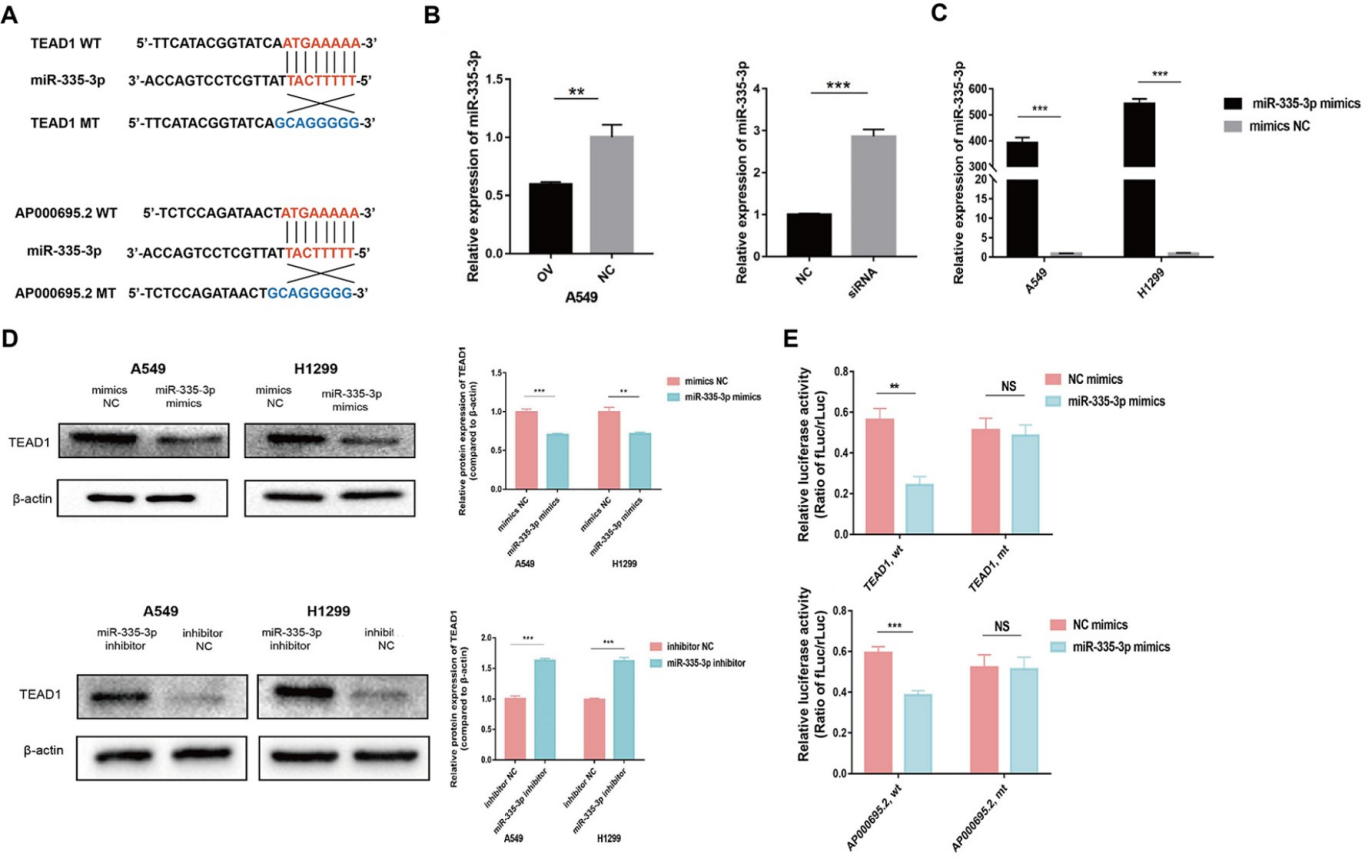
Figure 7 miR-335-3p acts as a competing endogenous RNA participating in the regulation of TEAD1 and AP000695.2 (A) The binding site of TEAD1 and miR-335-3p and the binding site of miR-335-3p and AP000695.2. (B) qPCR experiment showed that the expression of miR-335-3p in A549 cells was decreased after upregulating AP000695.2 expression. The expression of miR-335-3p in H1299 cells was increased after AP000695.2 was downregulated. (C) The expression of miR-335-3p in A549 and H299 cells was increased after the transfection of miR-335-3p mimics. (D) Western blot analysis showed that the protein expression of TEAD1 was decreased after the transfection with miR-335-3p mimics, and increased after the transfection with miR-335-3p inhibitor, compared with the negative control group. (E) Dual-luciferase reporter assay indicated that miR-335-3p has binding sites with TEAD1 and AP000695.2. **P<0.01, ***P<0.001. NS, no significance.

The miRDB database ( http://mirdb.org/) predicted the miRNA targeted binding between AP000695.2 and TEAD1. Further screening of the RNAhybrid ( https://bibiserv.cebitec.uni-bielefeld.de/rnahybrid/) and TargetScan databases ( https://www.targetscan.org/vert_80/) and considering the reliability of binding sites, miR-335-3p with 8 mer binding sites for AP000695.2 and TEAD1 binding was obtained. The mutant plasmid vectors were constructed and specific binding sites are shown in Figure 7A. qPCR analysis showed that the expression of miR-335-3p in A549 cells decreased after upregulating AP000695.2 expression ( Figure 7B). The expression of miR-335-3p in H1299 cells increased after AP000695.2 expression was downregulated ( Figure 7B). The negative regulatory relationship indicates that AP000695.2 and miR-335-3p can form the binding mechanism of ceRNA. After the transfection of miR-335-3p mimics, qPCR analysis showed that the expression of miR-335-3p in A549 and H1299 cells was higher than that in cells transfected with the mimics NC ( Figure 7C). Moreover, miR-335-3p mimics can significantly upregulate the expression of miR-335-3p. The miR-335-3p mimics and miR-335-3p inhibitor, and the corresponding NC were transfected into A549 and H1299 cells, respectively. Compared with the NC group, western blot analysis showed that the protein expression of TEAD1 decreased after the transfection of miR-335-3p mimics and increased after the transfection of miR-335-3p inhibitor ( Figure 7D), suggesting that TEAD1 and miR-335-3p possess a regulatory relationship. To prove the targeted binding relationship between miR-335-3p and TEAD1, wild-type TEAD1 3′UTR reporter plasmid (TEAD1, wt) and mutant TEAD1 3′UTR reporter plasmid (TEAD1, mt) were constructed as shown in Figure 7A. TEAD1, wt+NC mimics, TEAD1, wt+miR-335-3p mimics, TEAD1, mt+NC mimics, and TEAD1, mt+miR-335-3p mimics were cotransfected into HEK-293FT cells respectively. Dual-luciferase reporter assay showed that the fluorescence level of cells in TEAD1, wt+miR-335-3p mimics group was significantly lower than that in the TEAD1, wt+NC mimics group. Compared with that in the TEAD1, mt+NC mimics group, the fluorescence level of cells in the TEAD1, mt+miR-335-3p mimics group showed no significant difference ( Figure 7E). Moreover, a binding site between miR-335-3p and TEAD1 has been proved.

To prove the targeted binding relationship between miR-335-3p and AP000695.2, wild-type AP000695.2 reporter plasmid (AP000695.2, wt) and mutant AP000695.2 reporter plasmid (AP000695.2, mt) were constructed as shown in Figure 7A. AP000695.2, wt+NC mimics, AP000695.2, wt+miR-335-3p mimics, AP000695.2, mt+NC mimics, and AP000695.2, mt+miR-335-3p mimics were cotransfected into HEK-293FT cells respectively. The dual-luciferase assay showed that compared with that in the AP000695.2, wt+NC mimics cells, the fluorescence level of the AP000695.2, wt+miR-335-3p mimics group decreased significantly. Compared with that in the AP000695.2, mt+NC mimics group, the fluorescence level of the AP000695.2, mt+miR-335-3p mimics group showed no significant difference ( Figure 7E). Furthermore, a binding site between miR-335-3p and AP000695.2 was proved.

### miR-335-3p reverses the oncogenic function of AP000695.2

**Figure 8:**
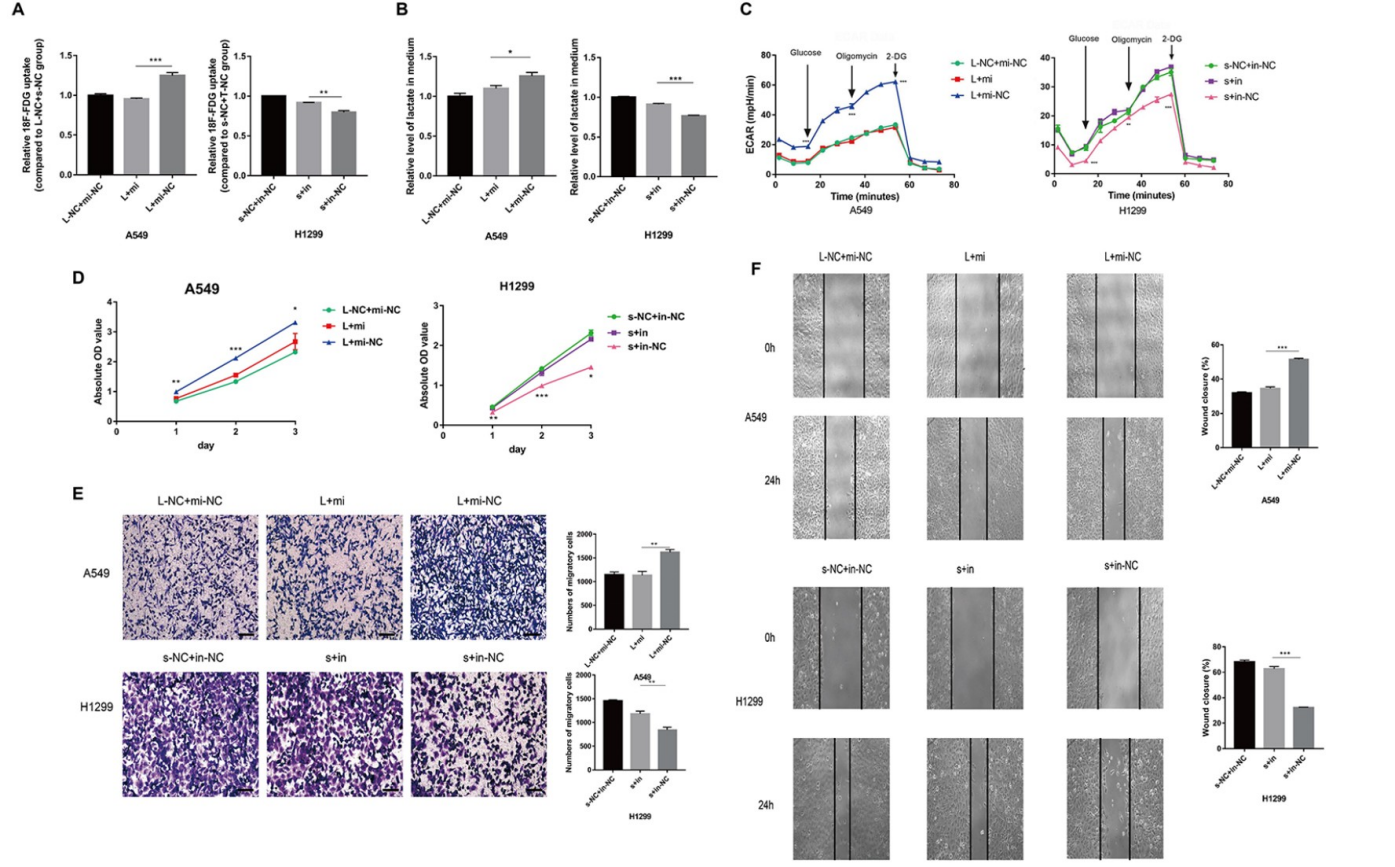
Figure 8 miR-335-3p reverses the oncogenic function of AP000695.2 (A) The relative amount of FDG uptake of the L+mi group (transfected with AP000695.2 overexpression plasmid and miR-335-3p mimics) was lower than that of the L+mi-NC (transfected with AP000695.2 overexpression plasmid and miR-335-3p mimics NC) group. The amount of FDG uptake in the s+in (transfected with AP000695.2 siRNA and miR-335-3p inhibitor) group was higher than that of the s+in-NC group (transfected with AP000695.2 siRNA and miR-335-3p inhibitor NC). (B) The amount of lactic acid production in the L+mi group was lower than that in the L+mi-NC group. The amount of lactic acid produced in the s+in group was higher than that in the s+in-NC group. (C) The level of glycolysis, glycolytic capacity and glycolytic reverse in the L+mi group were lower than those in the L+mi-NC group. The level of glycolysis, glycolytic capacity and glycolytic reverse in the s+in group were higher than those in the s+in-NC group. (D) The cell proliferation ability of the L+mi group was lower than that of the L+mi-NC group, and that of the s+in group was higher than that of the s+in-NC group on the 1st, 2nd and 3rd day after transfection. (E) The cell migration ability of the L+mi group was lower than that of the L+mi-NC group, and that of the s+in group was higher than that of the s+in-NC group (magnification × 200, scale bar: 100 μm). (F) The cell migration ability of the L+mi group was lower than that of the L+mi-NC group, and that of the s+in group was higher than that of the s+in-NC group (magnification × 100). *P<0.05, **P<0.01, ***P<0.001.

The relative amount of FDG uptake of the L+mi group (transfected with AP000695.2 overexpression plasmid and miR-335-3p mimics) was lower than that of the L+mi-NC group (transfected with AP000695.2 overexpression plasmid and miR-335-3p mimics NC). The amount of FDG uptake in the s+in group (transfected with AP000695.2 siRNA and miR-335-3p inhibitor) was higher than that of the s+in-NC group (transfected with AP000695.2 siRNA and miR-335-3p inhibitor NC) ( Figure 8A). The relative amount of lactic acid produced was calculated. The lactic acid content in the L+mi group was lower than that in the L+mi-NC group in A549 cells and the lactic acid content of the s+in group was higher than that in the s+in-NC group in H1299 cells ( Figure 8B). The level of glycolysis, glycolytic capacity and glycolytic reverse in the L+mi group was lower than those in the L+mi-NC group in A549 cells, while those in the s+in group was higher than those in the s+in-NC group in H1299 cells ( Figure 8C). These results indicated that miR-335-3p reverses the effect of AP000695 on glycolysis of LUAD. CCK-8 assay showed that the cell proliferation ability of the L+mi group was lower than that of the L+mi-NC group, and that of the s+in group was higher than that of the s+in-NC group ( Figure 8D). This indicated that miR-335-3p reversed the cell proliferation regulation of AP000695.2. Transwell assays showed that the cell migration ability of the L+mi group was lower than that of the L+mi-NC group, and that of the s+in group was higher than that of the s+in-NC group ( Figure 8E). Wound healing assays showed that the cell migration ability of the L+mi group was lower than that of the L+mi-NC group, and that of the s+in group was higher than that of the s+in-NC group ( Figure 8F), indicating that the effect of AP000695.2 may be mediated by miR-335-3p.

### Silencing of *AP000695*. *2* inhibits tumor growth and aerobic glycolysis *in vivo*

**Figure 9:**
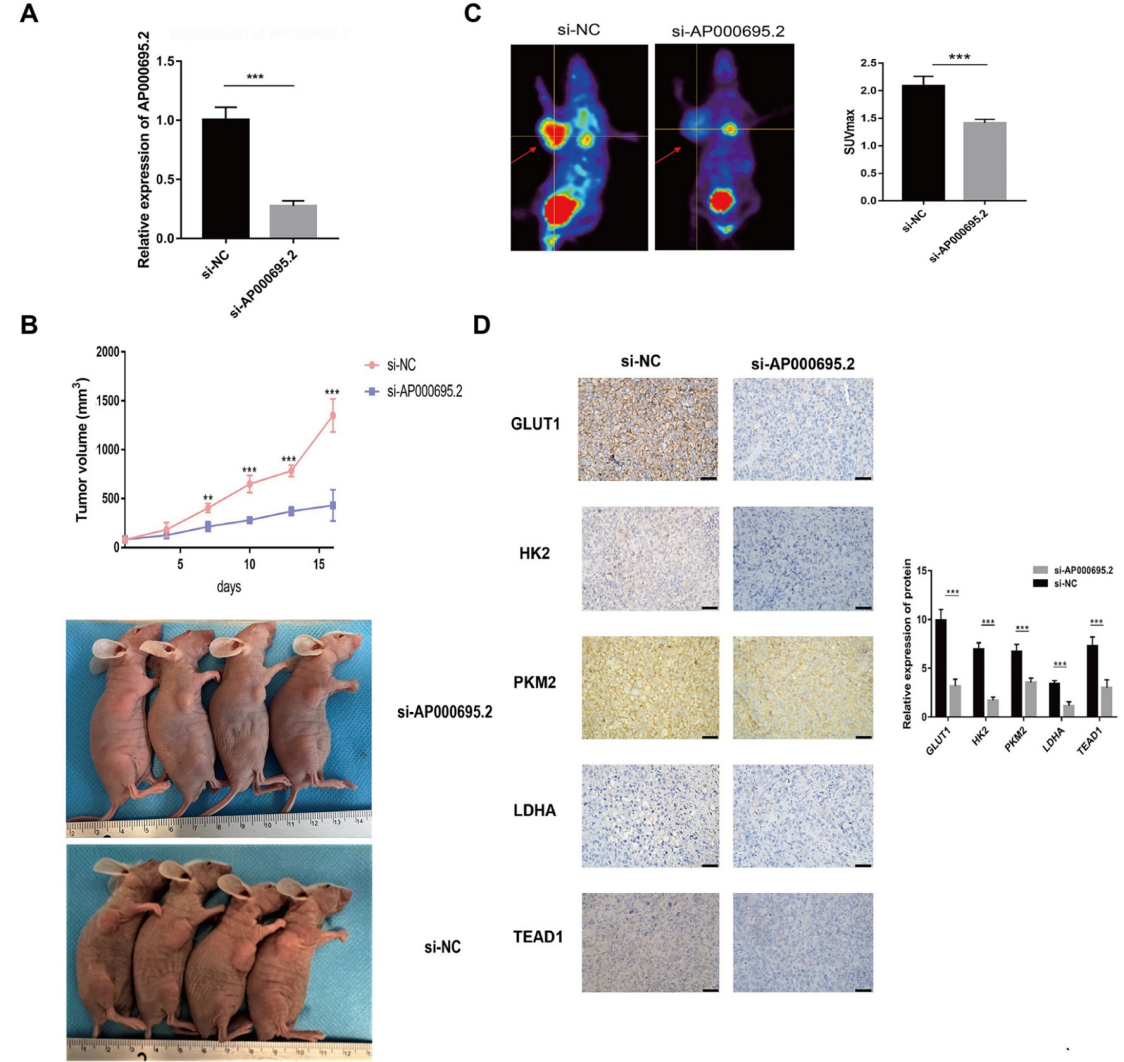
Figure 9 Silencing of AP000695.2 inhibits tumor growth and aerobic glycolysis *in vivo* (A) The qPCR experiment showed that the expression of si-AP000695.2 group is significantly lower than that of negative control group. (B) The tumor volumes at different time points showed that from the 6th day after injection, compared with the si-NC group, the si-AP000695.2 significantly inhibited the growth of transplanted tumor. (C) The uptake of 18F-FDG by tumor was decreased after the expression of AP000695.2 was decreased. The picture on the left showed the micro-PET images. The right picture showed the quantitative analysis results of SUVmax. (D) Immunohistochemistry (IHC) staining showed that the expression levels of GLUT1, HK2, PKM2, LDHA and TEAD1 in si-AP000695.2 group were lower than those in si-NC group. (magnification × 400, scale bar: 100 μm). **P<0.01, ***P<0.001.

After the subcutaneously transplanted tumors were successfully constructed, they were divided into si-NC and si-AP000695.2 groups according to the injected drugs ( *n* = 4). qPCR experiment showed that AP000695.2 expression in the si-AP000695.2 group was significantly lower than that of negative control group ( Figure 9A). Dynamic monitoring was performed on tumor growth and the tumor volumes at different time points suggested that from the 7th day after injection, compared with si-NC, si-AP000695.2 significantly inhibited the transplanted tumor growth ( Figure 9B). ^18^F-FDG micro-PET imaging was used to investigate its effect on tumor glycolysis. The uptake of ^18^F-FDG by tumors was expressed by SUVmax. Based on the injected drugs, the animals were divided into si-NC and si-AP000695.2 groups. The SUVmax of the si-AP000695.2 group decreased significantly ( Figure 9C). Moreover, IHC staining showed that the expression levels of GLUT1, HK2, PKM2, LDHA and TEAD1 in the si-AP000695.2 group were lower than those in the si-NC group, with a statistically significant differences ( Figure 9D, *t*=9.961, 14.07, 7.307, 8.555, 6.647).

## Discussion

The pathogenesis of NSCLC is complex, involving the synergy of multiple pathways. Moreover, the treatment targeting lncRNAs has broad prospects in NSCLC [20]. The wide application of bioinformatics analysis provides a new direction and technical means for identifying biomarkers. By analyzing LUAD data in TCGA database, we found that the novel lncRNA-AP000695.2 is closely related to glycolysis. Its gene is located in the forward strand of chrome 21: 36445731-36532408, and the Ensembl version number is ENSG00000233818.1. It has two transcripts. The transcript ID is ENST00000428667.1 (located in chrome 21: 36445731-36532408, with a length of 715 bp) and ENST00000454980.1 (located in chrome 21: 36445822-36460752, with a length of 649 bp).

*In vivo* and *in vitro* experiments were conducted to further explore the effects of AP000695.2 on glycolysis, proliferation ability, migration ability and EMT process of LUAD. Compared with that in normal lung epithelial cells, AP000695.2 was found to be highly expressed in seven NSCLC cell lines (four LUAD cell lines, and three NSCLC cell lines), as revealed by qPCR. Moreover, the overexpression plasmid was transfected into A549 cells with the lowest expression, and the downregulation experiment was performed in H1299 cells with the highest expression. After cellular localization, AP000695.2 was found to be expressed in both the cytoplasm and nucleus. Therefore, two technologies, siRNA and ASO, were selected for downregulation. The qPCR results showed that the knockdown efficiency of siRNA was higher. Therefore, siRNA was used in the follow-up experiment.

From the perspective of glycolysis, AP000695.2 was verified to promote the expression of glycolysis-related proteins by western blot analysis, which was also consistent with the results of bioinformatics analysis. Meanwhile, FDG uptake and lactic acid production assays further verified the hypothesis that AP000695.2 promotes the glycolysis of LUAD. The research by Wang *et al*. [21] confirmed that the cell proliferation and migration of LUAD is inhibited after silencing of *AP000695* . *2*. Our study verified this view again and perfected the view that AP000695.2 promotes the proliferation and migration of LUAD cells from the perspective of upregulation by using CCK-8, EdU experiment, Transwell and wound healing assay. The expression levels of N-Cadherin and Vimentin were detected by western blot analysis, which confirmed that AP000695.2 promotes EMT in cells of LUAD.

Next, we studied the mechanism of AP000695.2 regulating glycolysis in LUAD. Through bioinformatics analysis, we found that there is a significant positive correlation between AP000695.2 and TEAD1. TEAD1 is the key regulator of Hippo signal pathway [ 22, 23], and several studies suggested that it functions as a transcription factor participating in the transcription of GLUT1 in laryngeal squamous cell carcinoma, breast cancer and T-cell lymphoma [ 24- 26]. In this study, a dual-luciferase reporter assay was used to verify this assumption. TEAD1 can upregulate the expression level of GLUT1 in LUAD cells by upregulating the expression of TEAD1. As a key protein for transporting glucose into cells, GLUT1 plays an important role in the regulation of glycolysis, and an increase in its expression can activate the pathway. Therefore, TEAD1 is suggested to promote glycolysis by activating GLUT1 transcription.

Cellular and animal models verified that AP000695.2 regulates glycolysis, proliferation and migration of LUAD by regulating TEAD1. FDG uptake and lactic acid production assays further verified this speculation. CCK-8 experiment verified that AP000695.2 regulates the proliferation of LUAD cells by regulating TEAD1, and Transwell and wound healing assays verified that AP000695.2 regulates the migration of LUAD cells by regulating TEAD1. Therefore, AP000695.2 serves as a ‘bridge’ to connect the Hippo signaling pathway with glycolysis.

Many studies have confirmed that hypoxia-inducible factor-1α (HIF-1α) is closely related to glycolytic reprogramming. The regulatory effect of AP000695.2 and TEAD1 on HIF-1α were also verified by bioinformatics analysis and western blot analysis. Sun *et al.* [27] proved that TEAD1 can regulate the transcription of HIF1A and further promote tumor glycolysis. Therefore, our study suggests that AP000695.2 promotes HIF-1α expression through TEAD1, thus promoting glycolysis. This needs to be verified by further research, and the focus of this study is on the direct regulation of glycolytic pathway by AP000695.2 through TEAD1.

Furthermore, the mechanism of interaction between AP000695.2 and TEAD1 was explored. Through database screening and qPCR verification, we found the potential action factor miR-335-3p. Shen *et al.* [28] found that long noncoding RNA LINC00518 contributes to proliferation and metastasis in lung adenocarcinoma via the miR-335-3p/CTHRC1 axis. Pu *et al*. [29] found that in NCI-H1975 lung adenocarcinoma cell line, miRNA-335-3p downregulates the expression of coatomer protein complex subunit β2 (COPB2) to inhibit cell proliferation, migration and invasion and promote cell apoptosis. Zhao *et al* . [30] found that upregulated lncRNA CASC9 promotes the progression of non-small cell lung cancer by inhibiting miR-335-3p and activating S100A14 expression. In addition, studies have confirmed that upregulation of miR-335-3p expression can inhibit the progression of acute myeloid leukemia [31], breast cancer [32] and osteosarcoma [33], and plays an important role in regulating tumor progression. The target binding relationships between AP000695.2 and miR-335-3p, TEAD1 and miR-335-3p was verified by dual-luciferase reporter assay. AP000695.2 was found to bind to TEAD1 through miR-335-3p, thus participating in the regulation of the malignant progression of LUAD. However, the role of miR-335-3p in the regulatory mechanism of AP000695.2 needs further validation.

Nevertheless, certain limitations exist in this study. Firstly, the expression of AP000695.2 was not verified at the tissue level. Secondly, the role of AP000695.2 in other biological behaviors of LUAD, such as immunity and autophagy, needs further study. Finally, the regulatory role and mechanism of AP000695.2 in other tumors are worth exploring.

**Figure 10:**
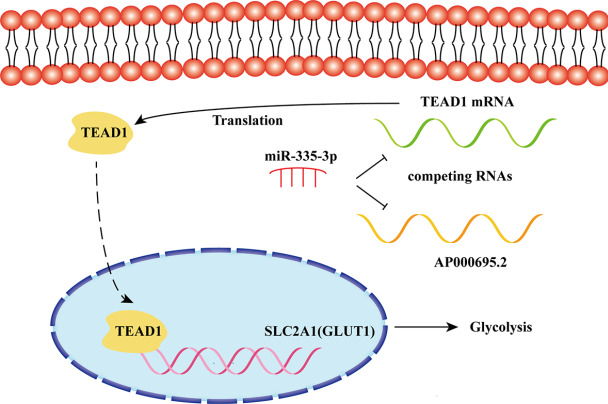
Figure 10 Schematic diagram of the mechanisms of AP000695.2/miR-335-3p/TEAD1/GLUT1 axis in the glycolysis of LUAD AP000695.2 is involved in the mechanism of LUAD through functioning as a ceRNA to competitively sponge miR-335-3p, thereby regulating the expression of TEAD1. Then TEAD1 promotes glycolysis of lung adenocarcinoma by promoting GLUT1 transcription.

In summary, this study demonstrated the role of lncRNA AP000695.2 in glycolysis of LUAD. Functional assays suggested that AP000695.2 functions as an oncogene by mediating glycolysis activation and promoting LUAD malignant progression via the miR-335-3p/TEAD1 axis ( Figure 10). Therefore, lncRNA AP000695.2 may serve as a potential biomarker and anti-tumor energy metabolism therapeutic target for LUAD.

## Supplementary Data

Supplementary data is available at *Acta Biochimica et Biophysica Sinica* online.

## Supporting information

461Supplementary_Table
