# Supplementary material for: LncRNA AP000695.2 promotes glycolysis of lung adenocarcinoma via the miR-335-3p/TEAD1 axis: The role of AP000695.2 in glycolysis of lung adenocarcinoma
Source: Acta Biochim Biophys Sin (Shanghai). 2023 Sep 18;55(10):1592–605. doi: 10.3724/abbs.2023227 (PMC10577454; doi:10.3724/abbs.2023227)
Supplement: 461Supplementary_Table [file 461Supplementary_Table.pdf]

Supplementary Table S1. Sequences of siRNA and miRNA used in this study

Gene Symbol	Sequence	
	sense（5'-3'）	antisense（5'-3'）
si-AP000695.2	GCAGGAAGAUGUACGUGAAdTdT	UUCACGUACAUCUUCCUGCdTdT
siRNA NC	UUCUCCGAACGUGUCACGdTdT	ACGUGACACGUUCGGAGAAdTdT
miR-335-3p mimics	UUUUUCAUUAUUGCUCCUGACC	GGUCAGGAGCAAUAAUGAAAAA
mimics nc	UCACAACCUCCUAGAAAGAGUAGA	UCUACUCUUUCUAGGAGGUUGUGA
miR-335-3p inhibitor	GGUCAGGAGCAAUAAUGAAAAA	

Supplementary Table S2. Sequences of plasmids used in dual-luciferase reporter assays

Name	Target sequence
GLUT1 promoter, wt	TGTAAACTGGTGCCATGAAGAAGGACCGTGATGGCCAGGCTTGGCCCTTACTCCCTCCA<br>TGACAATTTCAGACAGATGAGCAGGAGAGTGGCAGGGTGCAGGACTGGAGGACAAAA<br>GACCAGAGGAGATGGAGGCCCTGCAGACAGCAGGGGAGGGAAGGAGGATGAGGTGG<br>TGTAGACAGCGGAGTCAGCAGGACTTGGTCTGTCCAGGGGTGAGGAGAGAGGTGGCC<br>CAAGCTTTCCAGGCTGAGCATAGAGAGACCACAGGAATTGAAGCCTTGGGCAGGGGGG<br>TGCCCAGAATGCACCTGCCTTCTGTTTCAGGGCTCCATACTCAACCAAGGTGCCCCTCA<br>GCCAGAACTGGACACATTGCCAGAGAACTCCAGGGGTCATCTGGTCGGCTCAGCCTCC<br>CGGTTTCCGACAGACAGTAAGCCCACCATGACTCCCTCCATCGGGAAAGGGACCCTCA<br>AGACTTTGATCAGACCTTTGGACTGGGGGTCCTTCCCTTCACTGTGTCTCAAGTAAGGC<br>ACTGGTCTAGAGGAAATAAGGTGAAGTCAGATGATAGCAATACCACTACCAGTGCAGTG<br>GCACATGCCTGCACTCCCAGCTACTACGGAGGCTAAGGTGGGAGGATCACTTGAGCCC<br>AGGAGTTTGAGACCAGCATTTATTTATTTACTAACAGACTAAATAAATAAACCAACAATA<br>CCACCAACCAGTAAATGAGAACTTCCATGCTAAACACTTCATGAGCTTATCTGGAAATT<br>GCACCTCTCCTGATAGCAATGAGATTGGTGTGGGAATTCCATTTACTCATAAGAAGACA<br>GCCTCGGCCGGGCTCAGTGGCTCACGCCTGTAATCCCAGTACTTTGGGAGGCGAGGTG<br>GGTGGATCACGAGGTCAAGAGTTCAAGACCAGCCTGGCCAAAATGGTGAAACCCCGTC<br>TCTACTAAAAATAAAAATAATTAGCCAGGGGTGATAGCGGGCGCCTGTAATCCCAGCTA<br>CTCGGGAGGCTGAGGCAGAGAACTGCTTGAATCCGGGAGGCGGAGGTTACAGTGAGC<br>CGAGATCGTGCCGCTGCACTCCAGCCTGGGCGACAGAGCGAGACTCTGTCTGAGAAAA<br>AAAAAAATTAAAGAAAAAAACGAAAACAGCCTCACTGGCCAGGAACTCGCCCAGGAT<br>CGCGGCCGGGCCAGTATACAGTTGAGCTGGTTCAAACCCGAGGTCTATCATGTCCTTAA<br>TGTCCTATCATGTCAGTCAGTTAGCCTGCCTTTCATATTTTCCACACTTACAGGTCACCAT<br>AGACTCACCTGAACTGTGAGAAGTAGAGGAAGGCGTCCCAGAGGAGGTGATTTTAAAG<br>CCCAGGTTTGAGATGGGTATGCATTTCCAGGCAAGAGAGCCCGGCCGGTGCAAAAGCA<br>AGGCTTGGCTCACAAACGGGAATGTGGGGGGTGGAGACAGGGAAGGGAGAAGTCAAT<br>CCCTGGGCAAGACCTCCTGGAGTCTCTCTAACAAATAAATACTACTTTCCATGCAGTAG<br>ACGCTGTTCTAAACACTTTACAAATATTAACTCACTTGGTCTTTCTACAACCCTACGAGG<br>TGGTACTGTTACTATCCCTAGTGCACCGAAGTCACCCAGCGGCCGAGTGAGAACTCCAG<br>TCCAGCTTTCCACCCGCTACTCCGCGCATCCCAGCTTGCCTTACAGCCGGGTACCGGCT<br>CCACCATTTTGCTAGAGAAGGCCGCGGAGGCTCAGAGAGGTGCGCACACTTGCCCTGA<br>GTCACACAGCGAATGCCCTCCGCGGTCCCAACGCAGAGAGAACGAGCCGATCGGCAG<br>CCTGAGCGAGGCAGTGGTTAGGGGGGGCCCCGGCCCCGGCCACTCCCCTCACCCCCTC<br>CCCGCAGAGCGCCGCCCAGGACAGGCTGGGCCCCAGGCCCCGCCCCGAGGTCCTGCC<br>CACACACCCCTGACACACCGGCGTCGCCAGCCAATGGCCGGGGTCCTATAAACGCTAC<br>GGTCCGCGCGCTCTCTGGC
GLUT1 promoter, mt	TGTAAACTGGTGCCATGAAGAAGGACCGTGATGGCCAGGCTTGGCCCTTACTCCCTCCA<br>TGACAATTTCAGACAGATGAGCAGGAGAGTGGCAGGGTGCAGGACTGGAGGACAAAA<br>GACCAGAGGAGATGGAGGCCCTGCAGACAGCAGGGGAGGGAAGGAGGATGAGGTGG

	TGTAGACAGCGGAGTCAGCAGGACTTGGTCTGTCCAGGGGTGAGGAGAGAGGTGGCC<br>CAAGCTTTCCAGGCTGAGCATAGAGAGACCAAGCCTTGGGCAGGGGGGTGCCCTGCCT<br>TCTGTTTCAGGGCTCCATACTCAACCAAGGTGCCCCTCAGCCAGAACTGGACACATTGC<br>CAGAGAACTCCAGGGGTCATCTGGTCGGCTCAGCCTCCCGGTTTCCGACAGACAGTAA<br>GCCCACCATGACTCCCTCCATCGGGAAAGGGACCCTCAAGACTTTGATCAGACCTTTGG<br>ACTGGGGGTCCTTCCCTTCACTGTGTCTCAAGTAAGGCACTGGTCTAGAGGAAATAAGG<br>TGAAGTCAGATGATAGCAATACCACTACCAGTGCAGTGGCACATGCCTGCACTCCCAGC<br>TACTACGGAGGCTAAGGTGGGAGGATCACTTGAGCCCAGGAGTTTGAGACCAGCATTT<br>ATTTATTTACTAACAGACTAAATAAATAAACCAACAATACCACCAACCAGTAAATGAGAA<br>CTTCCATGCTAAACACTTCATGAGCTTATCTGGAAATTGCACCTCTCCTGATAGCAATGA<br>GATTGGTGTGTTACTCATAAGAAGACAGCCTCGGCCGGGCTCAGTGGCTCACGCCTGTA<br>ATCCCAGTACTTTGGGAGGCGAGGTGGGTGGATCACGAGGTCAAGAGTTCAAGACCAG<br>CCTGGCCAAAATGGTGAAACCCCGTCTCTACTAAAAATAAAAATAATTAGCCAGGGGTG<br>ATAGCGGGCGCCTGTAATCCCAGCTACTCGGGAGGCTGAGGCAGAGAACTGCTTGAAT<br>CCGGGAGGCGGAGGTTACAGTGAGCCGAGATCGTGCCGCTGCACTCCAGCCTGGGCGA<br>CAGAGCGAGACTCTGTCTGAGAAAAAAAAAAAATTAAAGAAAAAAACGAAAACAGCCT<br>CACTGGCCAGGAACTCGCCCAGGATCGCGGCCGGGCCAGTATACAGTTGAGCTGGTTC<br>AAACCCGAGGTCTATCATGTCCTTAATGTCCTATCATGTCAGTCAGTTAGCCTGCCTTTC<br>ATATTTTCCACACTTACAGGTCACCATAGACTCACCTGAACTGTGAGAAGTATCCCAGA<br>GGAGGTGATTTTAAAGCCCAGGTTTGAGATTTCCAGGCAAGAGAGCCCGGCCGGTGCA<br>AAAGCAAGGCTTGGCTCACAGGGGTGGAGACAGGGAAGGGAGAAGTCAATCCCTGGG<br>CAAGACCTCCTGGAGTCTCTCTAACAAAGCAGTAGACGCTGTTCTAAACACTTTACAAA<br>TATTAACTCACTTGGTCTTTCTACAACCCTACGAGGTGGTACTGTTACTATCCCTAGTGC<br>ACCGAAGTCACCCAGCGGCCGAGTGAGAACTCCAGTCCCCGCTACTCCGCTTGCCTTA<br>CAGCCGGGTACCGGCTCCACCATTTTGCTAGAGAAGGCCGCGGAGGCTCAGAGAGGTG<br>CGCACACTTGCCCTGAGTCACACAGCGAATGCCCTCCGCGGTCCCAACGCAGAGAGAA<br>CGAGCCGATCGGCAGCCTGAGCGAGGCAGTGGTTAGGGGGGGCCCCGGCCCCGGCCA<br>CTCCCCTCACCCCCTCCCCGCAGAGCGCCGCCCAGGACAGGCTGGGCCCCAGGCCCCG<br>CCCCGAGGTCCTGCCCACACACCCCTGACACACCGGCGTCGCCAGCCAATGGCCGGGG<br>TCCTATAAACGCTACGGTCCGCGCGCTCTCTGGC
TEAD1, wt	TTGGCTTTAAAGTGAATTTATCTTTAGTATGATAGTTATATGAAAATTATAGGATTTGTGTG<br>CAGAGAATTTTTTTATAAAGTGCTTTGTAAAAAAAAAAAAAATGTATTCTAGCTTTTGCGG<br>TACATATGTGTGATAACTTTAATACCCATGACAGTTAAGTGCAATTATTTCATCACTCTAA<br>AAATGCTATTTTTGTGTCAGTTCCTGCAGGTGTTTTCATGTCTTTGCAAAGTGACACATT<br>TTGATGCCTTCTTGATAAAGTGGTAGACATTTTGTAGCTTTCTAGAAACTTTGTATTCATA<br>CGGTATCAATGAAAAATAAAGAAAATGAAAGTGTGGG
TEAD1, mt	TTGGCTTTAAAGTGAATTTATCTTTAGTATGATAGTTATATGAAAATTATAGGATTTGTGTG<br>CAGAGAATTTTTTTATAAAGTGCTTTGTAAAAAAAAAAAAAATGTATTCTAGCTTTTGCGG<br>TACATATGTGTGATAACTTTAATACCCATGACAGTTAAGTGCAATTATTTCATCACTCTAA<br>AAATGCTATTTTTGTGTCAGTTCCTGCAGGTGTTTTCATGTCTTTGCAAAGTGACACATT<br>TTGATGCCTTCTTGATAAAGTGGTAGACATTTTGTAGCTTTCTAGAAACTTTGTATTCATA<br>CGGTATCAGCAGGGGGTAAAGAAAATGAAAGTGTGGG
AP000695.2, wt	GAACTAGTCTTCCCTGAAATGTACCTGACTACAGCTAACATCATATCACATGATGAAAGA<br>CCGCGTTGTTTTCCCCTGAGGTTGGCAGTGCAGGAAGATGTACGTGAAATAATTCATGG<br>AAAAGTCACAGTGGCCTTGAAGTTCTCCAGATAACTATGAAAAACTACGTTAAGATGCT<br>ATTTTTCAACAAAATTCATTTTACCAACCTCAAAACGTATTTCCTCCGAAGAGGATACGC<br>ACTGACAAACAACACCCGAGTGTGGCATGCTAGCAGGAGCACTAGGCTGGGAGTCAA<br>GAGACTTAAATTCCACCCGGAAGCCACCACATGACCTTGGCATGAGCCCGTCACCAGG<br>CTCCTCAGCGTCCTCATCGCAGCACCAGG
AP000695.2, mt	GAACTAGTCTTCCCTGAAATGTACCTGACTACAGCTAACATCATATCACATGATGAAAGA<br>CCGCGTTGTTTTCCCCTGAGGTTGGCAGTGCAGGAAGATGTACGTGAAATAATTCATGG<br>AAAAGTCACAGTGGCCTTGAAGTTCTCCAGATAACTGCAGGGGGCTACGTTAAGATGCT<br>ATTTTTCAACAAAATTCATTTTACCAACCTCAAAACGTATTTCCTCCGAAGAGGATACGC<br>ACTGACAAACAACACCCGAGTGTGGCATGCTAGCAGGAGCACTAGGCTGGGAGTCAA<br>GAGACTTAAATTCCACCCGGAAGCCACCACATGACCTTGGCATGAGCCCGTCACCAGG<br>CTCCTCAGCGTCCTCATCGCAGCACCAGG

Supplementary Table S3. Prediction results of binding sites between *TEAD1* and *GLUT1* promoter

Matrix ID	Name	Score	Relative score	Sequence ID	Start	End	Strand	Predicted sequence
MA0090.2	TEAD1	9.71142	0.922217260346	SLC2A1	1416	1425	-	CACATTCCCG
MA0090.2	TEAD1	8.9742	0.907027452075	SLC2A1	790	799	+	GGAATTCCAT
MA0090.2	TEAD1	8.26747	0.892466039612	SLC2A1	1309	1318	-	CGCCTTCCTC
MA0090.2	TEAD1	8.16047	0.890261368527	SLC2A1	1499	1508	+	TACTTTCCAT
MA0090.2	TEAD1	7.62171	0.879160554181	SLC2A1	1355	1364	-	TGCATACCCA
MA0090.3	TEAD1	10.62	0.875338612796	SLC2A1	1414	1426	-	CCACATTCCCGTT
MA0090.2	TEAD1	7.26167	0.87174228075	SLC2A1	291	300	-	TGCATTCTGG
MA0090.2	TEAD1	7.20536	0.870582008214	SLC2A1	1492	1501	+	TAAATACTAC
MA0090.2	TEAD1	6.87162	0.86370573108	SLC2A1	1659	1668	+	CGCATCCCAG
MA0090.2	TEAD1	6.69954	0.860160059803	SLC2A1	1638	1647	+	AGCTTTCCAC
MA0090.2	TEAD1	6.48914	0.855824983485	SLC2A1	261	270	-	TCAATTCCTG
MA0090.1	TEAD1	10.2131	0.850775215188	SLC2A1	1414	1425	-	CACATTCCCGTT
